# Evaluation of 3D-printed bolus for radiotherapy using megavoltage X-ray beams

**DOI:** 10.1007/s12194-023-00727-0

**Published:** 2023-06-09

**Authors:** Chunsu Zhang, Will Lewin, Ashley Cullen, Daniel Thommen, Robin Hill

**Affiliations:** 1 Institute of Medical Physics, School of Physics, The University of Sydney, Sydney, NSW 2006 Australia; 2 Arto Hardy Family Biomedical Innovation Hub, Chris O’Brien Lifehouse, Camperdown, NSW 2050 Australia; 3 Department of Radiation Oncology, Chris O’Brien Lifehouse, Missenden Rd, Camperdown,Sydney, NSW 2050 Australia

**Keywords:** 3D printing, Radiotherapy, Bolus, Percentage depth dose (PDD), Surface doses, Megavoltage X-ray beams

## Abstract

A radiotherapy bolus is a tissue-equivalent material placed on the skin to adjust the surface dose of megavoltage X-ray beams used for treatment. In this study, the dosimetric properties of two 3D-printed filament materials, polylactic acid (PLA) and thermoplastic polyether urethane (TPU), used as radiotherapy boluses, were investigated. The dosimetric properties of PLA and TPU were compared with those of several conventional bolus materials and RMI457 Solid Water. Percentage depth-dose (PDD) measurements in the build-up region were performed for all materials using 6 and 10 MV photon treatment beams on Varian linear accelerators. The results showed that the differences in the PDDs of the 3D-printed materials from the RMI457 Solid Water were within 3%, whereas those of the dental wax and SuperFlab gel materials were within 5%. This indicates that PLA and TPU 3D-printed materials are suitable radiotherapy bolus materials.

## Introduction

In radiotherapy, it is necessary to deliver the prescribed dose to the tumor while minimizing the dose to the surrounding healthy tissue [1]. The most common radiotherapy modality uses megavoltage X-ray beams produced by linear accelerators. One of the important dosimetric properties of megavoltage X-ray beams is the build-up effect, in which the dose increases rapidly with depth within the first few millimeters [2]. The maximum dosage occurs several centimeters deep in the body, with a typical maximum dose at 1.5 cm for 6 MV X-ray beams, resulting in the skin-sparing effect [3, 4].

In some clinical situations, such as treating post-mastectomy breast or head and neck cancer [1, 2, 5, 6], the dose at or close to the skin must be close to the prescribed dose. In these situations, the dose at the skin is increased by placing a bolus on the skin surface [7]. A bolus is a radiologically tissue-equivalent material whose properties, such as mass density and relative electron density, are similar to those of soft tissue [8, 9]. An ideal patient bolus should be conformable to the surface, non-toxic, readily available, cost-effective, easily adjustable to the desired thickness, and comfortable for the patient [1, 8]. Traditional bolus materials, such as dental wax and SuperFlab gel, are widely used in clinical practice. However, their densities and thicknesses can vary depending on the method of placement and variations in the manufacturing process [10]. They are mostly handmade; therefore, they are subject to errors during fabrication [11].

The use of 3D printing in medical applications, such as the production of personalized boluses, has advanced quickly [12–17]. To date, research has shown that 3D-printed bolus can conform well to the body surface anatomy of the patient [18] and reduce the gap between the surface and the bolus [3, 19]. Previous studies have shown that 3D-printed bolus makes appropriate and adequate dosing of the skin or subcutaneous tissue possible [4], thereby improving the quality and reproducibility of dosimetry plans [20].

Despite the many potential advantages of integrating 3D-printing technology into radiotherapy, many challenges remain unresolved [21]. However, new materials for 3D filaments have been commercially released that may be suitable for use as radiotherapy boluses. For example, polylactic acid (PLA) is widely used but has the disadvantage of low pliability, which makes it quite rigid when it is being positioned on the patient. A small number of studies have used thermoplastic polyether urethane (TPU), which has significant flexibility compared with the rigid materials used in the previous studies, allowing for improved patient comfort, easy placement, and good shape retention [13, 22, 23].

In this study, the dosimetric properties of two 3D-printed materials, PLA and TPU, were evaluated for use as radiotherapy boluses with megavoltage X-ray beams. They were compared with several conventional bolus materials, including dental wax, SuperFlab gel, and RMI457 Solid Water.

## Materials and methods

In this study, several bolus materials were tested for their dosimetric properties, as listed below:

- Dental wax (INVESTO, Sydney, Australia),
- SuperFlab gel material bolus (Radiation Products Design Inc., Albertville, MN, USA)
- Bilby3D brand Flexible TPU filament (Bilby3D, Melbourne, Australia)
- Raise3D Premium PLA Filament (RAISE3D, Irvine, California, USA)
- RMI457 Solid Water (Gammex/RMI, Middleton, WI, USA).

Dental wax and SuperFlab are materials used as clinical radiotherapy boluses. RMI457 Solid Water was selected as the reference material, because it has been shown to have very good radiological water equivalence over a wide range of X-ray beam energies, within 1% [24, 25]. The compositions and nominal physical densities of the materials used are listed in Table 1 as provided by the vendors and published data [7, 8, 12, 35, 36].

**Table 1:** Composition and physical density data of the materials used

Materials	Known composition information	Physical density (g/cm^3^)
Dental wax	60% Paraffin Wax, 25% Carnuba Wax, 10% Ceresin and 5% Beeswax	1.02
SuperFlab gel material	Proprietary composition	1.03
RMI457 solid water	Fractional weights: H(0.0809), C(0.6722), N(0.024), O(0.1984), Cl(0.0013) and Ca(0.0232)	1.04
TPU pre-print filament	C_4_H_4_O_4_	1.14
PLA pre-print filament	(C_3_H_4_O_2_)_n_	1.20

**Fig. 1:**
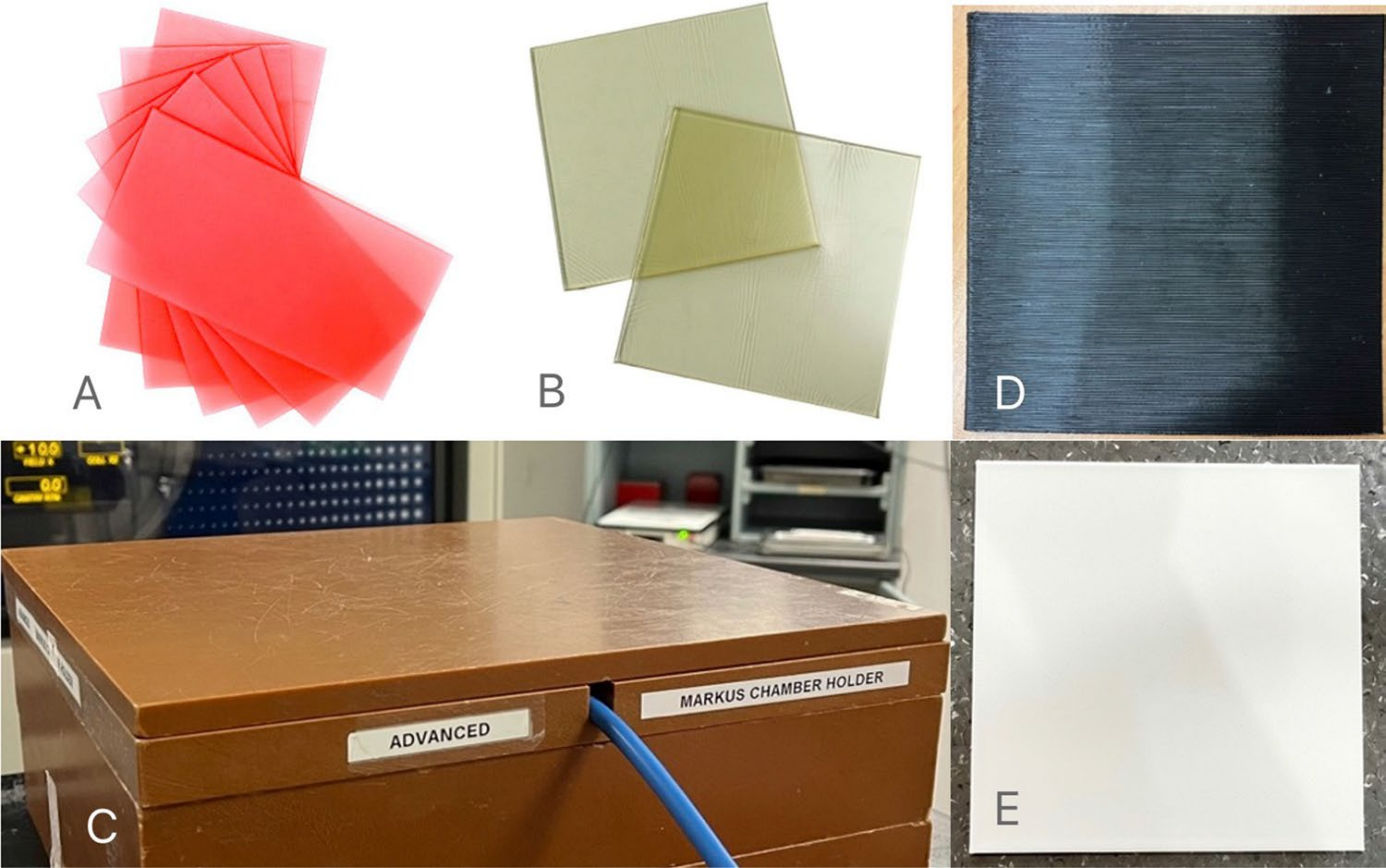
A. Dental wax, B. SuperFlab gel, C. RMI457 Solid Water, D. TPU slab, and E. PLA slab

The blocks of RMI457 Solid Water had dimensions of 30 × 30 cm, with thicknesses ranging from 0.1 to 5.0 cm. The specific thicknesses of the dental wax and SuperFlab gel material were as provided by the manufacturers. The thickness of a single piece of dental wax was 1.5 mm, and the SuperFlab slabs had two set thicknesses of 0.5 and 1 cm. The TPU and PLA filaments were used to 3D print the TPU and PLA slabs with different nominal thicknesses ranging from 1 to 10 mm (Fig. 1).

### 3D-printing process

The 3D-printed bolus slabs were designed using Fusion 360 software (Autodesk, San Francisco, CA, USA), exported to a 3MF file, and sliced for printing with IdeaMaker (RAISE3D, Irvine, CA, USA). The TPU and PLA slabs were printed using a Pro2 Plus 3D printer (RAISE3D, Irvine, CA, USA), which is a large-format fused filament fabrication 3D printer with dual print heads. In the 3D-printing process, TPU and PLA were printed with 100% infill to maximize the density and homogeneity of the printed slabs.

In 3D printing, many parameters control where and how the material is deposited and the density of the printed parts to build different objects during the printing procedure. The key parameters of 3D-printed TPU and PLA are listed in Table 2. To achieve the maximum print density, a concentric infill was selected, enabling small gaps to be filled by the IdeaMaker 3D slicer. Additionally, the slabs were printed standing vertically at the end, similar to how a chest wall bolus should be printed. However, the 1- and 2-mm-thick slabs needed to be printed horizontally (flat), because they would not self-support; therefore, a rectilinear infill was used for these. Many parameters can be adjusted or optimized to enhance the print quality or speed, each of which can affect bolus uniformity adversely. In addition to the visual observation of print quality, it is important to confirm that the features of the printed part meet the specific requirements of its intended use [20, 26]. Before dose measurement, the actual thickness of the printed slabs was measured using a caliper with an accuracy of 0.1 mm.

**Table 2:** Key parameters of 3D-printed TPU and PLA slabs

Parameter	TPU (1, 2 mm slabs)	TPU (5, 10 mm slabs)	PLA (1 and 2 mm slabs)	PLA (5 and 10 mm slabs)
Nozzle diameter (mm)	1.0	1.0	0.4	0.4
Layer height (mm)	0.7	0.7	0.35	0.35
Extrusion width (mm)	1.2	1.2	0.48	0.48
Number of shells	1	1	1	1
Infill pattern	Rectilinear	Concentric	Rectilinear	Concentric
Printing orientation	Horizontal	Vertical	Flat	Vertical
Infill density (%)	100	100	100	100
Extruder temperature (℃)	220	220	205	205
Bed temperature (℃)	60	60	60	60
Extruder speed (mm/s)	15	15	20	20
Cooling fan speed (%)	100	100	100	100
Retraction (mm)	8.0	8.0	1.5	1.5
Flow rate (%)	95	95	95	95

### Radiation dose measurement setup

An Advanced Markus plane-parallel ionization chamber (PTW, Freiburg, Germany, model number: 34045) was used for all dose measurements. This particular ionization chamber was chosen, because it is suitable for measuring the surface and build-up region doses of megavoltage photon beams [27–30]. This ionization chamber has an active sensitive volume with a diameter of 5 mm and a height of 1 mm.

The ionization chamber was positioned in a dedicated piece of RMI457 Solid Water placed on the central axis of the field, with 10 cm of Solid Water set underneath to ensure adequate backscattering. Solid Water slabs of different thicknesses were placed on top of the Advanced Markus ionization chamber to enable doses to be measured in the build-up region. For all the measurements, the ionization chamber was connected to a PTW UNIDOS electrometer (PTW, Freiburg, Germany) with an applied bias voltage of + 300 V.

The percentage depth-dose (PDD) data measured for the 6 MV beam were obtained using a Clinac 6EX linear accelerator (Varian Medical Systems, Palo Alto, CA, USA), whereas the measurements for the 10 MV beam were obtained using a TrueBeam linear accelerator (Varian Medical Systems, Palo Alto, CA, USA). The two linear accelerators were calibrated using a fixed source-to-surface distance (SSD) of 100 cm with a field size of 10 × 10 cm and 100 monitor units (MUs), equivalent to 1.000 Gy, at the depth of the maximum dose in water following the IAEA TRS398 Code of Practice [31]. For the measurements, the gantry and collimator angles were set to 0.0°, and the beam dose rates were set to 600 MU/min.

### 3D-printed slab uniformity test

**Fig. 2:**
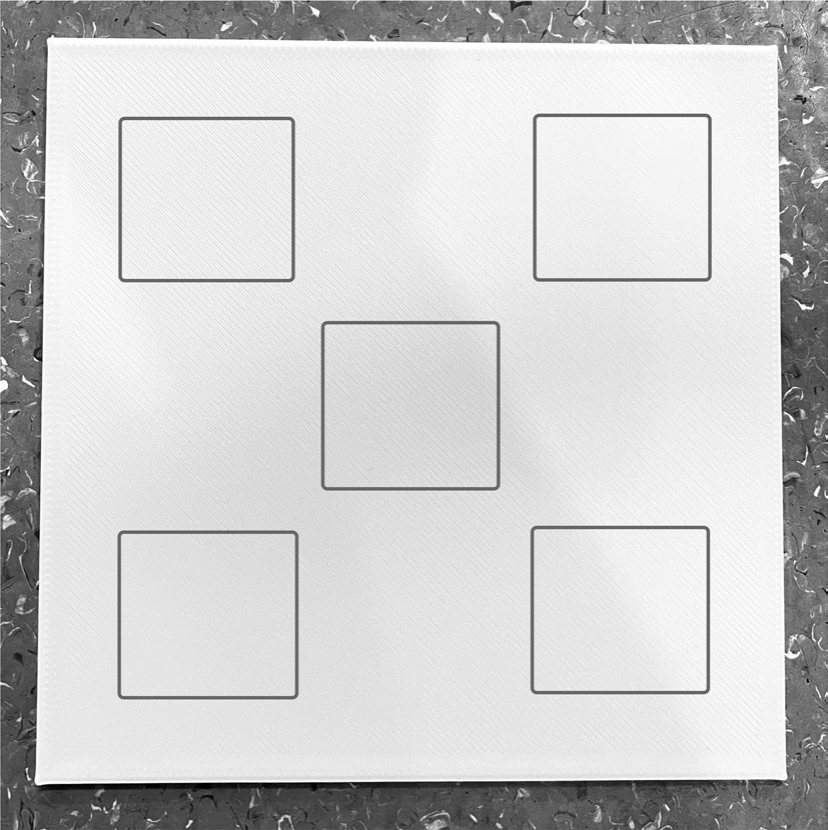
Five areas on a slab for uniformity test

The uniformity of the 3D-printed bolus slabs with thicknesses of 1–10 mm was confirmed by measuring the dose through different areas of the individual slabs. The TPU and PLA slabs printed in this project were 14 × 14 cm in size. To measure the uniformity of the bolus, each slab was divided into five separate locations, each with an area of 3 × 3 cm in the middle and four corners, as shown in Fig. 2. The SSD was set to 100 cm after the slabs were placed, the ionization chamber was placed below the slabs, and 3 × 3 cm fields were irradiated on the 6EX machine with 200 MU of 6 MV photon irradiation for uniformity measurements. Each location was irradiated twice under the same conditions, and the results were averaged. The measurements were compared with the center values for all slabs.

### PDD measurements

The Advanced Markus ionization chamber was also used for the PDD measurements in the build-up region for all the materials studied. The depth of the PDD measurement in this study was the thickness of the material above the ionization chamber during the actual measurement. Dose measurements were performed under the same conditions for the 6 and 10 MV photon beams, with a field size of 10 × 10 cm and 200 MUs per reading. Each location was irradiated at least twice under the same conditions, and the results were averaged. The results were plotted using MATLAB (MathWorks, Natick, MA, USA).

For the PDD measurements, as many points as possible were measured in the build-up region up to the depth of the maximum dose (D_max_). The depths of D_max_ were 15 and 23 mm for the 6 MV and 10 MV X-ray beams, respectively. For the dose measurements in RMI457 Solid Water, TPU, and PLA, data could be collected at 1 or 2 mm intervals. However, wax was only available in multiples of 1.5 mm thickness, whereas the SuperFlab material bolus was only available in 5- and 10-mm-thick sheets. The depth doses measured in the RMI457 Solid Water were taken as the reference dose values for all comparisons.

## Results

### Actual thickness of 3D-printed slabs

The measured thicknesses of the 3D-printed TPU and PLA slabs are listed in Table 3. There is a small difference between the measured and designed thicknesses of the printed slabs. The results show that the maximum thickness variation of the 3D-printed TPU is 0.2 mm/mm, and the maximum variation of the 3D-printed PLA is 0.1 mm/mm.

**Table 3:** Measured thicknesses of the 3D-printed TPU and PLA slabs

Nominal thickness (mm)	Thickness of TPU slabs (mm)	Thickness of PLA slabs (mm)
1	1.2	1.1
2	2.2	2.2
5	5.0	5.0
10	10.0	10.3

### Uniformity of 3D-printed slabs

In Table 4, the transmission difference values of the dose measurements performed under the same conditions for different areas of each 3D-printed TPU and PLA slab are listed, using the middle area of each sheet as the reference for comparison of the results. In this way, the consistency of the density of the printed objects can be determined. The data comparison shows that the dose difference for all TPU slabs is within 0.8%. The PLA slab measurements with the same conditions show results less than or equal to 1.1%. These results show there is good uniformity for all 3D-printed sheets.

**Table 4:** Uniformity results for each 3D-printed slab

Thickness (mm)	TPU dose non-uniformity (%)	PLA dose non-uniformity (%)
1	0.5	1.1
2	0.2	0.2
5	0.8	0.2
10	0.3	0.1

### PDD measurements

#### 6 MV X-ray beam

**Fig. 3:**
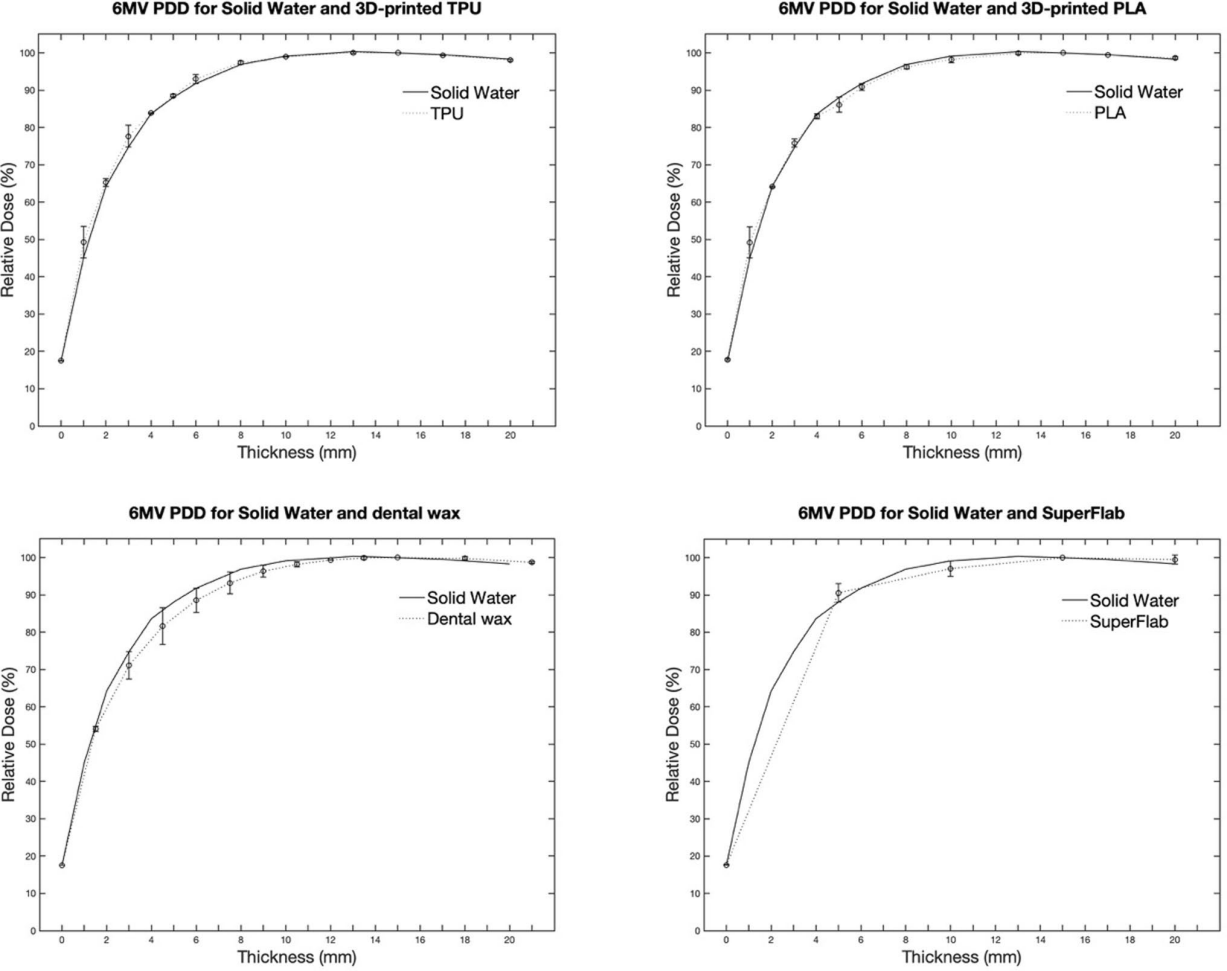
PDD comparison with RMI457 Solid Water of the four materials (TPU, PLA, dental wax, and SuperFlab gel) for 6 MV X-ray beams

Figure 3 shows the PDDs in the build-up region for the different materials, as measured for the 6 MV X-ray beam. The error bars are the standard deviation of the readings taken during the measurements. All the measurements are normalized to the maximum dose point (depth = 1.5 cm). At a gantry angle of 0°, the PDD increases from 15% to approximately 40% for all materials within 1 mm of the build-up region for 6 MV and a 10 × 10 cm field size. In the build-up region, all materials exhibit similar depth-dose characteristics.

Over the range of depths tested, the doses measured with the TPU and PLA agreed well with those measured with RMI457 Solid Water, with a maximum difference of 3%. The TPU exhibits a maximum difference of 2.9% at a depth of 1 mm, and a maximum difference of 3% for PLA occurs at a depth of 1 mm. A maximum deviation of 4.7% for wax occurred at a depth of 4 mm and 3% for SuperFlab at a depth of 5 mm, which are slightly more than those of TPU and PLA.

#### 10 MV X-ray beam

**Fig. 4:**
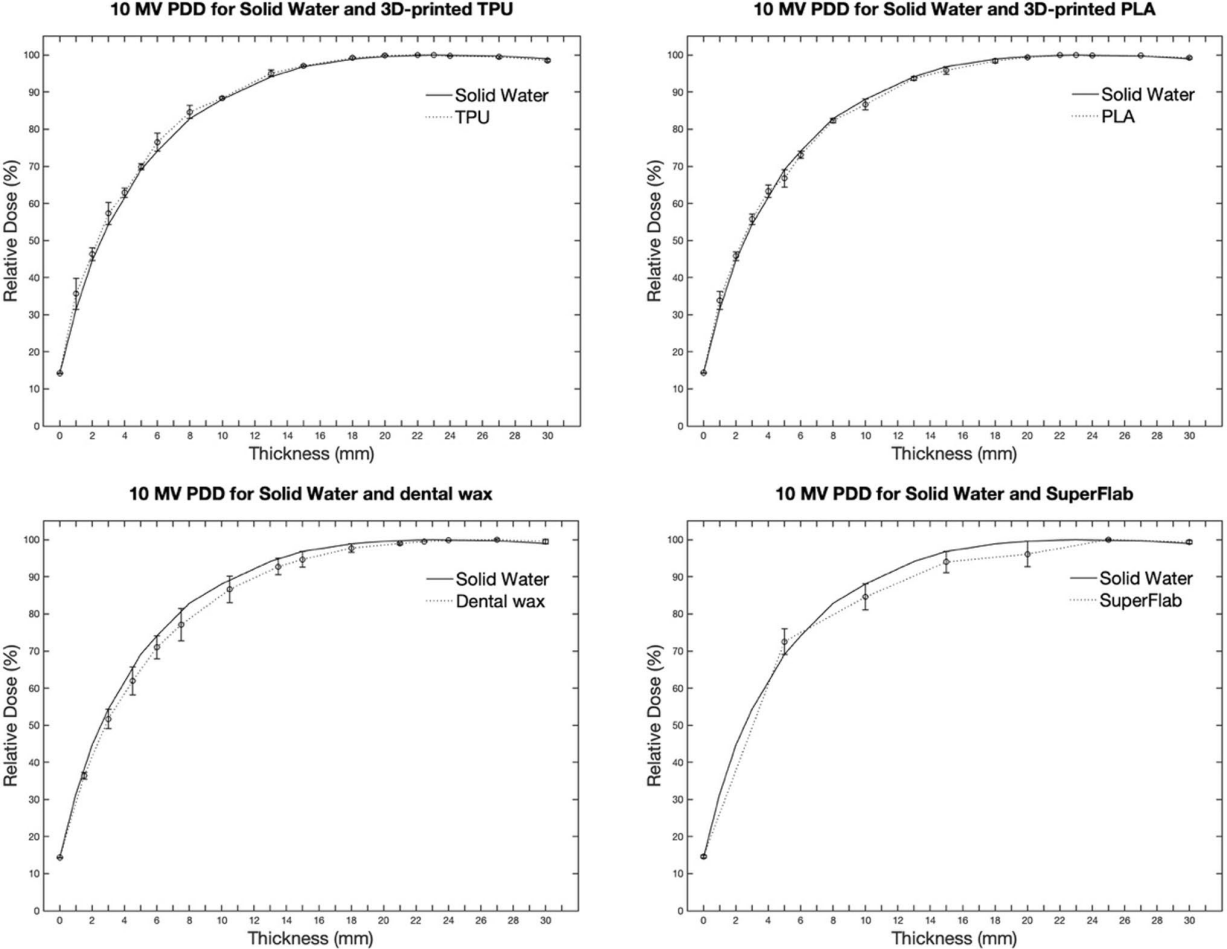
PDD comparison with RMI457 Solid Water of the four materials (TPU, PLA, dental wax, and SuperFlab gel) for 10 MV X-ray beams

The PDDs measured for the 10 MV X-ray energy were normalized to the dose at the depth of D_max_, as shown in Fig. 4. The doses measured in the TPU were slightly greater than those measured in the Solid Water. In comparison, the doses measured in the PLA and dental wax were slightly lower than those in the Solid Water. For SuperFlab, the dose at 5 mm was slightly higher, but the dose at 10 mm was slightly lower than the dose for the Solid Water.

The dose differences between TPU and RMI457 Solid Water were within 2% over the range of depths measured. The difference between PLA and Solid Water was within 3%, with a maximum difference of 2.4% at a depth of 1 mm. The maximum differences for the dental wax and SuperFlab gel were 4.4% at 7 mm for wax and 3.5% at 10 mm for SuperFlab.

## Discussion

A customized 3D-printed radiotherapy bolus has significant benefits in ensuring accurate delivery of therapeutic doses to the skin surface. For irregular body surfaces, it is possible to develop boluses with variable thicknesses according to body surface variations, which can provide a clinical advantage over conventional boluses.

A 3D-printed bolus can be printed accurately, and the individualized fit of the bolus to the irregular skin surface of the patient reduces air gaps [4]. In this study, it was demonstrated that the bolus could be printed to an accuracy of 0.2 mm, and this appeared to be consistent with other research results [12, 32]. The thickness of a conventional bolus, as supplied by the manufacturer, does not always match the specified optimal thickness for clinical applications.

PLA and TPU are different materials, and although they have been shown to have radiological properties similar to those of RMI-457 Solid Water, they are not necessarily direct substitutes for radiotherapy boluses. The most important factors to be considered are flexibility and stiffness, which are some of the main factors that distinguish TPU from PLA. In the authors’ experience, TPU is an excellent material for fabricating flexible, bendy, elastic, rubbery, or soft 3D-printed parts, whereas PLA is much more rigid. These physical properties may indicate which material should be used for the radiotherapy bolus and may vary according to the treatment site and size of the bolus.

Both TPU and PLA shrink slightly during the cooling time after manufacturing, with TPU shrinking between 0.4 and 1.4% [33] and PLA shrinking between 0.8 and 3% [34]. Despite the small shrinkage rates, it is important to confirm that they still conform to the body surface structure of the patient after cooling. The TPU and PLA used in this study have heat deflection temperatures and melting points that are much higher than the ambient temperature during use or storage.

One limitation of this study was that the SuperFlab slabs had fixed thicknesses of 5 and 10 mm sheets; therefore, the selection of measurement points in this study was limited compared with the other materials tested. This means that, for clinical scenarios in which other thicknesses of bolus may be required, a compromise may be needed in bolus thickness selection.

An important consideration for clinical radiotherapy departments is that printing a large bolus requires a significant amount of time. Because the printing process may take more than 1 day, an accurate bolus requires minimal user intervention during fabrication and a high-quality 3D printer with a low failure rate. In addition, radiological testing of the 3D-printed bolus is necessary to ensure that it meets the specific dose requirements of individual patients. Therefore, time is also needed for quality assurance of the fabricated bolus, such as accurate dimensions and homogeneity of the printed material.

## Conclusion

The dosimetric properties of the 3D-printed TPU and PLA slabs were investigated and compared with those of conventional bolus materials and Solid Water. In this study, the measured PDDs of different materials were compared. The experimental results obtained with 6 and 10 MV X-ray beams show that the dosimetric properties of the 3D-printed materials investigated are closer to those of the reference Solid Water than the conventional SuperFlab gel. Therefore, both TPU and PLA can be considered suitable materials for creating 3D-printed boluses for radiotherapy.
